# *Pseudomonas aeruginosa* Strain 91: A Multifaceted Biocontrol Agent against Banana Fusarium Wilt

**DOI:** 10.3390/jof9111047

**Published:** 2023-10-25

**Authors:** Jin Xie, Pratiksha Singh, Yanhua Qi, Rajesh Kumar Singh, Qijian Qin, Cheng Jin, Bin Wang, Wenxia Fang

**Affiliations:** 1 Institute of Biological Sciences and Technology, Guangxi Academy of Sciences, Nanning 530007, China; xiejin@gxas.cn (J.X.); singh.pratiksha23@gmail.com (P.S.); qiyanhua@gxas.cn (Y.Q.); qqij@gxas.cn (Q.Q.); jinc@im.ac.cn (C.J.); 2 Guangxi Key Laboratory of Sugarcane Genetic Improvement, Sugarcane Research Institute, Guangxi Academy of Agricultural Sciences, Nanning 530007, China; rajeshsingh999@gmail.com; 3 State Key Laboratory of Mycology, Institute of Microbiology, Chinese Academy of Sciences, Beijing 100101, China

**Keywords:** antagonism activity, *Fusarium oxysporum* f. sp. *cubense*, Banana Fusarium wilt, plant growth-promoting traits, *Pseudomonas aeruginosa*

## Abstract

Banana Fusarium wilt (BFW), caused by the soil-borne fungus *Fusarium oxysporum* f. sp. *cubense* (*Foc*), poses significant threats to banana cultivation. Currently, effective control methods are lacking, and biological control has emerged as a possible strategy to manage BFW outbreaks. In this investigation, 109 bacterial strains were isolated from the rhizospheric soil surrounding banana plants in search of potent biological agents against *Foc*. Strain 91 exhibited the highest antifungal activity against the causal agent of *Foc* and was identified as *Pseudomonas aeruginosa* through 16S rRNA gene sequencing and scanning electron microscopy (SEM). Elucidation of strain 91’s inhibitory mechanism against *Foc* revealed a multifaceted antagonistic approach, encompassing the production of bioactive compounds and the secretion of cell wall hydrolytic enzymes. Furthermore, strain 91 displayed various traits associated with promoting plant growth and showed adaptability to different carbon sources. By genetically tagging with constitutively expressing GFP signals, effective colonization of strain 91 was mainly demonstrated in root followed by leaf and stem tissues. Altogether, our study reveals the potential of *P. aeruginosa* 91 for biocontrol based on inhibition mechanism, adaptation, and colonization features, thus providing a promising candidate for the control of BFW.

## 1. Introduction

Banana (*Musa* spp.) is a significant fruit crop within the Musaceae family, extensively cultivated in developing countries. Banana Fusarium wilt (BFW), mainly caused by *Fusarium oxysporum* f. sp. *cubense* tropical race 4 (*Foc* TR4), stands as one of the most devastating diseases, significantly hampering global banana production [1,2]. The pathogen was first identified in Southeast Asia in the early 1990s. By the early 21st century, an endemic outbreak of *Foc* TR4 had extended to substantial areas in Australia’s northern territories, China, Indonesia, and Malaysia [3]. The fungus is highly stress-resistant and can endure in soil for up to 30 years, resulting in widespread orchard devastation [4]. *Foc* initially infects the roots and then invades the plant’s vascular tissue, resulting in wilting and eventual plant death [3].

Chemical control methods are not only costly and inefficient but also pose potential risks to public health and the environment. Conversely, biological control, specifically the use of plant growth-promoting rhizobacteria (PGPR), offers a promising alternative to chemical control while circumventing the challenges associated with conventional plant protection systems [5]. In recent years, biological control has garnered significant attention in various pathosystems. The discovery of microorganism-based biological control agents for banana diseases is highly expected [6]. Effective colonization of the rhizosphere and the ability to maintain a substantial population are considered essential prerequisites for the efficacy of PGPR [7]. Thus, it is important to find indigenous biocontrol strains with not only the efficiency of suppressing local pathogenic strains but also the adaptability to local conditions and the ability to compete with in situ microorganisms [8].

The rhizosphere is the region where soil particles come into contact with plant roots, constituting a dynamic and highly complex microbial ecosystem [9]. This environment facilitates intricate interactions between plants and microorganisms, resulting in a distinctive ecosystem characterized by carbon and water cycling, as well as the sequestration of nutrients and minerals. The examination of plant–microbe interactions and the diverse metabolites produced through their co-metabolism is of significant interest. These metabolites serve various functions, such as acting as energy sources and signaling molecules [10,11]. Rhizobacteria inhabiting the rhizosphere have been shown to synthesize a broad array of beneficial compounds [12]. Several studies have demonstrated that certain rhizobacteria possess antimicrobial properties against causal agents of plant diseases [13,14,15,16]. Healthy plant rhizosphere soil serves as a valuable source of PGPR [17], which readily colonize plant roots and promote plant development through various mechanisms [18]. These mechanisms include phosphate solubilization, the production of plant growth regulators, nitrogen fixation, ethylene metabolism, and the indirect enhancement of disease resistance via the production of antimicrobial metabolites or siderophores that inhibit harmful microorganisms [19]. Consequently, PGPR is gaining recognition as an environmentally friendly alternative to agrochemicals for sustainable agriculture. PGPR offers several advantages. It can serve as a substitute for non-leguminous agricultural fertilizers, enhance nutrient uptake by banana plants, and efficiently colonize banana root surfaces, where microbial cell density surpasses that in the root hair growth zone [20].

Antifungal rhizobacteria have received extensive attention for the biological control of plant diseases. The *Pseudomonas* genus encompasses over a hundred species [21], with many native to plant rhizosphere, endosphere, and phyllosphere environments, establishing commensal relationships therein. Some *Pseudomonas* strains have found application as plant inoculants due to their ability to mitigate the detrimental effects of specific phytopathogens, thereby promoting plant growth and health [22,23]. Several *Pseudomonas* species, including *P. aeruginosa*, *P. putida*, *P. chlororaphis*, *P. syringe*, and *P. fluorescens*, are well-recognized for their capacity to enhance plant development and suppress various plant diseases [23,24]. In Chinese biofertilizers, the primary strains are *Bacillus subtilis*, *Paenibacillus mucilaginosus*, *B. amyloliquefaciens*, *B. licheniformis*, and *B. megaterium* [25]. Conversely, various countries employ *Pseudomonas*-based biofertilizers, including *P. fluorescens* and *P. putida* in Vietnam, *P. fluorescens* in Cuba and Sri Lanka, *P. striata* in India, and *P. azotoformans* and *P. chlororaphis* in Sweden [26]. In recent years, a diverse array of *Pseudomonas* spp. strains has been explored as antagonists against *Foc*, offering new insights into the biological management of BFW. Most research in this field has focused on *P. fluorescens*, while our understanding of *P. aeruginosa* strains remains limited.

In this study, we isolated *Pseudomonas aeruginosa* strain 91 from banana rhizospheric soil due to its antagonistic activity against *Foc*. We investigated its inhibition mechanism, PGP traits, and colonization properties, affirming the potential of strain 91 as a promising candidate for the biological management of BFW.

## 2. Materials and Methods

### 2.1. Collection of Soil Samples and Bacteria Isolation

Rhizospheric soil samples were randomly collected from six different banana fields in Long’an county, Nanning city, Guangxi zhuang autonomous region, China. In each field, three healthy banana plants, five months old, were selected, and soil adhering to their roots was carefully gathered upon uprooting, with subsequent removal of root debris through a 2 mm mesh sieve. Samples were stored at 4 °C and processed within 24 h of collection. To isolate bacteria, 10 grams of soil from each sample were individually mixed with 90 mL of saline water (0.85% NaCl) in separate flasks. These suspensions were placed on an orbital shaker at 100 rpm and incubated at 32 °C for 1 h. After incubation, the soil suspensions of all six samples were serially diluted up to 10^−6^ and 100 µL of each dilution was spread on the Nutrient Agar (NA), Luria-Bertani (LB) Agar, Yeast Mannitol Agar, and Pikovskaya’s Agar media (Table S1) and incubated for 2 days at 32 °C. A total of 109 bacterial colonies were purified for further studies. All pure cultures were preserved in 20% glycerol at −80 °C.

### 2.2. Antifungal Assay of Isolated Bacterial Strains

The antifungal activity of all isolated strains was evaluated against the pathogenic fungus *Foc* (isolated by our group and stored in our lab) using a dual-culture method on Potato Dextrose Agar (PDA):Nutrient Agar (NA) at 1:1 (Table S1). Initially, a 10 μL aliquot of pathogen spores at a concentration of 10^6^ spores/mL was aseptically dispensed at the central region of the Petri dish. Subsequently, the experimental strain was inoculated at an approximate distance of 1 cm from the plate’s edge. Incubation was then conducted at 28 °C for 7 days, or until complete mycelial growth was detected on the control plate. The inhibition percentage was calculated using the following formula: [(R_1_ − R_2_)/R_1_] × 100, where R_1_ represents the radial growth of the fungal pathogen in the control plate and R_2_ represents the radial growth of the fungal pathogen in the presence of the tested strain [27]. 

Subsequently, strain 91 was cultured in nutrient broth (NB) medium for 7 days in an orbital incubator shaker at 80 rpm at 32 °C. The culture filtrate was obtained by centrifugation (10,000 rpm, 10 min, 20 °C) and subsequent filter sterilization (0.2 µm pore size). Spores of *Foc* were suspended in sterile distilled water and diluted into a concentration of 10^5^ spores·mL^−1^. A volume of 0.1 mL of diluted spores was spread on PDA Petri dishes (90 mm diameter). Wells of 5 mm diameter were punched into the inoculated plates and filled with 100 and 150 µL of cell-free culture filtrate. The plates were kept at 28 °C for 7 days. 

### 2.3. Extraction of Crude Metabolites from Strain 91

Strain 91 was subjected to solid-state fermentation yeast extract medium (YAG) plates, with a total fermentation volume of 9 L at a temperature of 25 °C for 9 days. Bacterial cultures were cut into small pieces and extracted exhaustively using a solution of ethyl acetate (EtOAc):methanol (MeOH):acetic acid (HAc) at a ratio of 80:15:5 (*v*/*v*/*v*) three times to generate a crude extract. The extracts were dissolved in water and first extracted three times with EtOAc, then extracted three times with n-butanol. A total of 15.779 g of EtOAc extract and 3.127 g of n-butanol extract was obtained after condensation and evaporation with a rotary evaporator. 

Antifungal activity against *Foc* was assessed using the disk diffusion method [28]. The crude extracts were dissolved in methanol and added to paper disks (6 mm in diameter) at the concentrations of 2, 4, and 6 mg/disk, respectively. The dried paper disks were applied onto the surface of the assay plates seeded with *Foc* spores for 48 h at 28 °C. Each treatment was repeated three times. The diameter of the fungal inhibition zone was then determined.

### 2.4. Fractionation of Crude Extracts

The EtOAc extracts (15.779 g) were subjected to a silica gel G column (200–300 mesh, Qingdao Marine Chemical Factory, Qingdao, China) using petroleum ether: EtOAc at 90:10, 80:20, 70:30, 60:40 and chloroform (CHCl_3_): MeOH at 90:10 and 0:100 gradient solvent system to produce 11 fractions (Fr.1–Fr.11). After the antifungal activity assay, Fr.7 was purified by Sephadex LH-20 (MeOH, 2 × 100 cm, 1 mL/min, Amersham Pharmacia, Uppsala, Sweden) to produce 5 fractions (Fr.7.1–Fr.7.5) for further activity assay.

### 2.5. Morphology and Interaction Study of Isolate 91 with Foc

Scanning electron microscopy (SEM, HITACHI, SU8100, Tokyo, Japan) was used to investigate the morphology and interaction of isolate 91 with *Foc*. For testing interactions, a 5 mm mycelial disc was taken from the interaction region and transferred to glass coverslips. After washing with phosphate buffer (PB) (0.1 M; pH 7.4), fixing with glutaraldehyde (2.5 %) and PB (0.1 M; pH 7.4) for 2 h at room temperature, and subsequently post-fixing by OsO_4_ (1%) in PB (0.1 M; pH 7.4), the coverslips were desiccated in a critical point dryer (EMITECH model K850 Hitachi, Tokyo, Japan). The samples were placed on stubs and sputter-coated with 10 nm Au before being examined using SEM. *Foc* without interaction was used as a control. 

### 2.6. Enzymatic Assay of Cell Wall-Degrading Enzymes Produced by Isolate 91

To test whether strain 91 produced cell wall-degrading enzymes, the isolate was streaked on an NA plate and incubated for 36 h at 32 °C. A single pure bacterial colony was inoculated in NB medium (10 mL) and cultured for 36 h at 32 °C in an orbital shaker (180 rpm) and centrifuged for 5 min at 4 °C and at 12,000 rpm to obtain the supernatant. The supernatant was filtered to detect the activities of chitinase (kit no. MM1062O1), cellulase (kit no. MM91502O1), β-1,3 glucanase (kit no. MM91504O1), and protease (kit no. MM1206O1) using enzyme-linked immune sorbent assays (ELISA) (Wuhan Colorful Gene Biological Technology Co., Ltd., Wuhan, China).

### 2.7. Plant Growth-Promoting Characteristics of Isolate 91

Isolate 91 was cultivated on NB medium for 36 h at 32 °C in an orbital shaker (120 rpm) to examine PGP characteristics. Standard techniques for estimating qualitative and quantitative production of siderophore [29,30], ammonia [31,32], and P-solubilization [33,34] were used. A qualitative technique was used to determine the production of hydrogen cyanide (HCN) [35]. The synthesis of indole-3-acetic acid (IAA) was measured using a spectrophotometer at 530 nm in the presence (0.5%) and absence of tryptophan in the medium [36]. 

The 1-Aminocyclopropane-1-carboxylate (ACC) deaminase activity of isolate 91 was measured using the [37] method with nitrogen-free Dworkin and Foster (DF) salts minimal medium [38] (Table S1). Medium lacking ACC was utilized as a negative control, whereas medium with (0.2% *w*/*v*) ammonium sulfate (NH_4_)_2_SO_4_ or 3 mM ACC was utilized as a positive control. Growth of 91 was detected after 5 days of incubation at 32 °C.

### 2.8. BIOLOG^(R)^ GENIII Phenotypic Assay

BIOLOG Phenotype Micro-Array^TM^ GENIII plate (Biolog Inc., Hayward, CA, USA) was used to investigate the prospective carbon (C) consumption profile of isolate 91. After cultivation on NA medium at 32 °C for 48 h, isolate 91 was suspended in inoculation fluid (IF) to achieve a transmittance of 90–98%. A total of 100 µL of cell suspension was placed into each well of Micro Plate’s 96 wells and incubated at 35 °C for 48 h to produce the phenotypic fingerprint. During incubation, the wells experience enhanced respiration, allowing the cells to use various carbon sources to grow. The tetrazolium dye is reduced by increased respiration, resulting in purple color. Following incubation, readings were recorded using an automated BIOLOG^(R)^ Micro-Station Reader (Biolog Inc., Hayward, CA, USA)by the manufacturer’s recommendations.

### 2.9. GFP Tagging of Isolate 91 and Colonization in Banana Plantlets

Plasmid GFP-pPROBE-pTet^r^-TT encoding green fluorescent protein (GFP) was collected from Guangxi University, Nanning, China. Freshly cultivated 91 and *Escherichia coli* strain containing GFP-pPROBE-pTetr-TT plasmid were cultured in LB broth media and combined at 1:2 then kept in an orbital shaker (160 rpm) at 35 °C for 48 h. After the incubation, 100 µL of bacterial broth was dispersed on LB agar plates and left overnight to test for the presence of the tagged strain. Tagging was further confirmed by confocal laser scanning microscopy (CLSM). 

Tissue cultures of banana plantlets were collected from Guangxi Academy of Agricultural Sciences, Nanning, China, and washed with autoclaved distilled water before being incubated with bacteria. Plantlets were grown in a growth chamber at 30 °C in an autoclaved cylindrical glass container (V = 200 mL) with 50 mL of MS liquid medium (sucrose and basal salt mixture). The 500 μL tagged bacterial suspension (~2.0 × 10^5^ mL^−1^) was carefully transferred into the plantlets in bottles and then the bottles were placed back into a growth chamber set at 30 °C with a 14 h photoperiod and a photon flux density of 60 µ moL m^−2^ s^−1^. After 72 h of growth, plantlets were removed, cleaned with autoclaved water, and CLSM was performed to check the colonization of isolate 91. The root, stem, and leaf tissues of both inoculated and uninoculated banana plantlets were chopped into small pieces (50 to 150 µm) and placed on the bridge slide using a 10% (*v*/*v*) glycerol solution. The CLSM (Leica DMI 6000, Leica Microsystems, Mannheim, Germany) was used to detect all plant sections [39,40].

### 2.10. Identification of Isolate 91

To amplify the 16S rRNA gene in isolate 91, DNA was utilized as a template with primer pairs PA-F (AGAGTTTGATCCTGGCTCAG) and PH-R (AAGGAGGTGATCCAGCCGCA) [41], following PCR conditions of initial denaturation at 95 °C for 5 min, 30 cycles of denaturation at 95 °C for 1 min, annealing at 55 °C for 1 min, extension at 72 °C for 1 min, and final extension at 72 °C for 5 min. The amplified product was purified by a PCR purification kit (BioFlux, Hangzhou, China) and sequenced (Sangon Biotech, Shanghai, China).

### 2.11. Phylogenetic Study

The 16S rRNA gene sequence of 91 was compared to essential reference sequences in the NCBI GenBank database to confirm its identity and evolutionary relationships. ClustalW [42] was used to align the various sequences and the sequences were compared using the BlastN search tool (NCBI, USA, https://blast.ncbi.nlm.nih.gov/Blast.cgi, accessed on 15 February 2021). MEGA X (molecular evolutionary genetics analysis, 10.0.2) software was used to perform a phylogenetic analysis of isolate 91 [43].

## 3. Results

### 3.1. Isolation, Screening, and Identification of Effective Antagonistic Bacteria from Banana Rhizosphere

**Figure 1:**
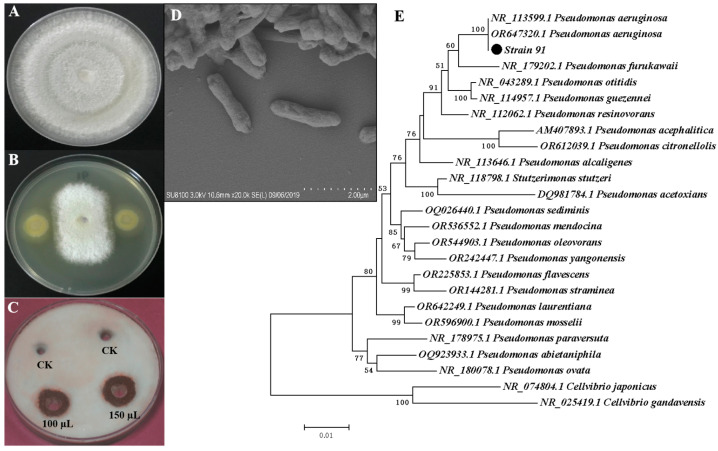
Screening and identification of isolate 91 for antifungal activity against *Fusarium oxysporum* f. sp. *cubense* (*Foc*). (**A**) *Foc* control plate after 7 days’ incubation; (**B**) dual-culture plate assay showing inhibition of *Foc* mycelia by isolate 91 after 7 days’ incubation; (**C**) cell-free fermentation supernatant displayed growth inhibition zone against *Foc* compared to control (CK); (**D**) scanning electron microscopy showing rod-shaped isolate 91; (**E**) phylogenetic tree showing isolate 91’s position compared to other *Pseudomonas* strains.

From six rhizospheric soil samples of banana plants, we isolated 109 bacterial strains. We evaluated all these isolates for their antifungal activity against *Foc*. Isolate 91 exhibited the highest antifungal activity, inhibiting 69% of *Foc* growth in the dual-culture plate assay (Figure 1A,B). Moreover, the cell-free fermentation supernatant of isolate 91 demonstrated inhibition of the *Foc* pathogen when compared to the control (Figure 1C). 

Isolate 91’s identification involved initial examination under light microscopy, revealing its motile, rod-shaped cells. This identification was further validated through scanning electron microscopy (SEM) (Figure 1D) and 16S rRNA sequencing analysis. The 16S rRNA sequence of isolate 91 was compared to nucleotide sequences in the NCBI GenBank database using the BlastN program. A phylogenetic tree was constructed using representative strains obtained from BlastN, confirming isolate 91 as *P. aeruginosa* (Figure 1E). The accession number OL658832 was assigned to *P. aeruginosa* 91 and submitted to the NCBI GenBank database.

### 3.2. Isolate 91 Displays a Multivariate Mode of Antagonism Mechanism

**Figure 2:**
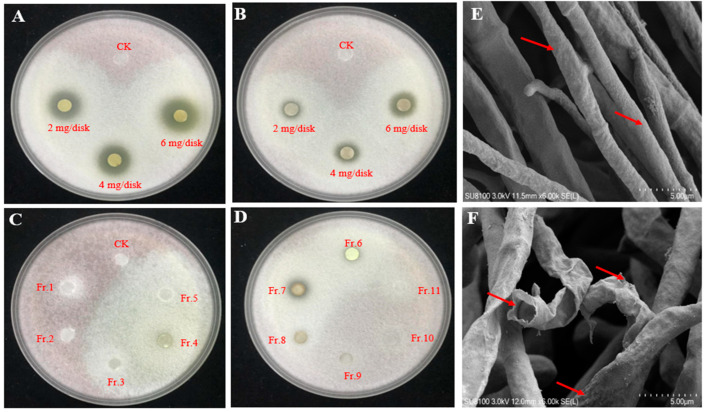
Isolate 91 secrets bioactive metabolites and cell wall-degrading enzymes for antagonistic activity against *Foc*. (**A**) EtOAc extract displayed inhibition activity at 2 mg/disk, 4 mg/disk, and 6 mg/disk, and CK is methanol. (**B**) n-butanol extract displayed inhibition activity at 2 mg/disk, 4 mg/disk, and 6 mg/disk, and CK is methanol. (**C**) Fr.1–Fr.5 of EtOAc extract displayed no inhibition activity, and CK is methanol. (**D**) Inhibition activity of Fr.6–Fr.11 of EtOAc extract. (**E**) *Foc* mycelia under SEM, red arrows showing straight, intact hyphae. (**F**) *Foc* mycelia under SEM after incubating with isolate 91, red arrows showing distorted, ruptured hyphae.

We initially hypothesized that the antagonistic activity of isolate 91 stemmed from its secondary metabolites. To investigate this, we conducted separate extractions of the solid fermentation material on YAG plates using ethyl acetate and n-butanol. The EtOAc extract, at concentrations of 2 mg/disk, 4 mg/disk, and 6 mg/disk, exhibited inhibition zone diameters of 11 mm, 14 mm, and 17 mm, respectively (Figure 2A). Conversely, the n-butanol extract, at concentrations of 2 mg/disk, 4 mg/disk, and 6 mg/disk, displayed inhibition zone diameters of 7 mm, 8 mm, and 9 mm, respectively (Figure 2B). These results indicate that both EtOAc and n-butanol extracts contain bioactive compounds capable of inhibiting the growth of *Foc*.

The EtOAc extract was further separated into 11 fractions (Fr.1–Fr.11), which were incubated at 1.5 mg/disk to detect inhibition activity. Among the 11 fractions, only Fr.7 displayed a transparent inhibition zone at 7 mm (Figure 2C,D). Further fractionation of Fr.7 was conducted and an activity test revealed a 7 mm inhibition zone of Fr.7.4 at 1.5 mg/disk. It was noticed that step-by-step purification of the EtOAc failed to show increased antifungal activity. Therefore, it is reasonable to speculate that instead of a single bioactive compound displaying antifungal activity, multiple components in the EtOAc extract exhibit synergistic antifungal effects.

Microorganisms can inhibit fungal growth directly by secreting various cell wall-degrading enzymes [44]. To determine whether isolate 91 produces these enzymes, we conducted an activity assay, which revealed cellulase activity at 451.23 ± 5.37 IU·mL^−1^, glucanase activity at 665.76 ± 10.84 IU·mL^−1^, protease activity at 143.56 ± 1.82 IU·mL^−1^, and chitinase activity at 495.43 ± 5.32 IU·mL^−1^ under in vitro conditions (Table 1). Additionally, SEM images showed distorted and ruptured *Foc* mycelia when incubated with isolate 91 (Figure 2F), confirming the secretion of cell wall-degrading enzymes by isolate 91 to disrupt the fungal cell wall. In conjunction with its multiple bioactive metabolites, isolate 91 exhibits a multifaceted antagonistic mechanism.

### 3.3. Isolate 91 Possesses Various Plant Growth-Promoting (PGP) Traits

**Figure 3:**
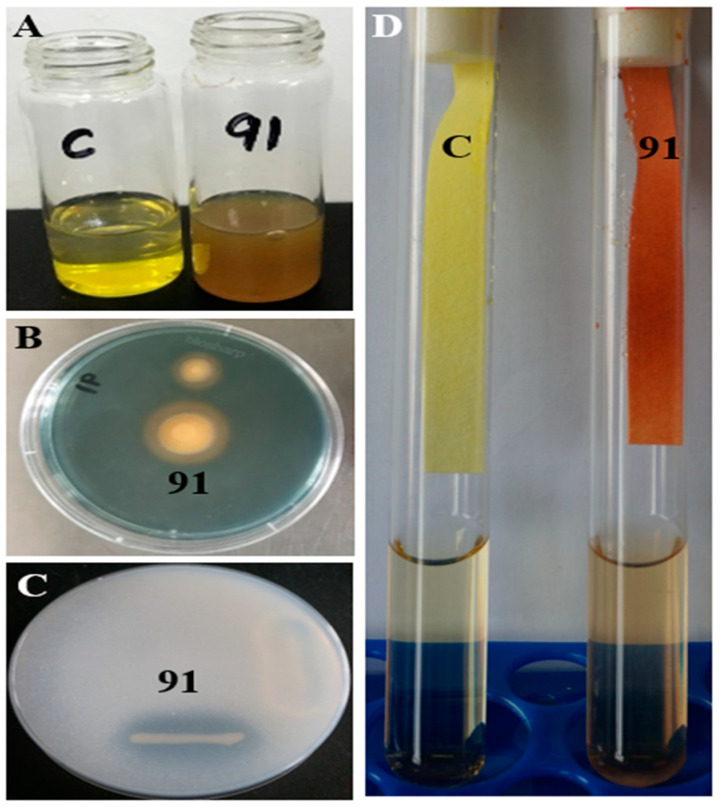
Plant growth-promoting traits of isolate 91. (**A**) Ammonia production on peptone water broth medium; (**B**) siderophore production on Chrome Azurol S Agar plate medium; (**C**) phosphate solubilization on Pikovskaya’s Agar plate medium; (**D**) HCN production in Luria-Bertani broth medium containing glycine.

PGP is an attractive trait for biocontrol microorganisms [45]. Isolate 91 exhibits a diverse range of PGP traits (Table 1 and Figure 3). It produces substantial levels of ammonia (5.53 ± 0.07 µmoL mL^−1^) (Figure 3A). On Chrome Azurol S Agar medium, it forms an orange halo zone, indicating siderophore production (85.21 ± 0.06 PSU) (Figure 3B). When grown on Pikovskaya’s Agar medium, it creates a clear zone, suggestive of phosphate solubilization capability (1.38 ± 0.03 PSI) (Figure 3C). Isolate 91 also exhibits a positive HCN production test (Figure 3D).

Isolate 91 exhibited significant IAA synthesis capacity, producing 175.96 ± 1.63 µg·mL^−1^ of IAA in a medium containing 0.5% tryptophan, while 49.53 ± 1.48 µg·mL^−1^ of IAA was generated in tryptophan-free media (Table 1). Furthermore, in DF-ACC medium with 3 mM ACC as the sole nitrogen source, isolate 91 displayed ACC deaminase enzyme activity by consuming ACC, yielding an estimated rate of 519.28 ± 6.58 nmoL α-ketobutyrate mg^−1^·h^−1^ after 48 h of incubation (Table 1). 

### 3.4. Isolate 91 Colonizes in All Tissues of Banana Plants

**Figure 4:**
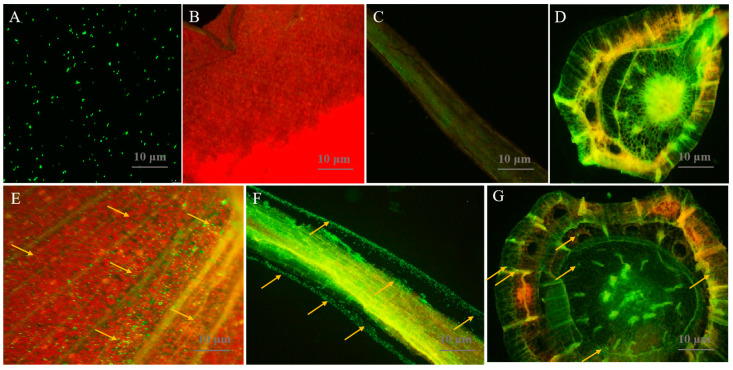
Confocal laser scanning microscopy (CLSM) presenting morphology and colonization of *P. aeruginosa* 91 in banana plants. (**A**) GFP-tagged 91 strain; (**B**–**D**) are tissues of leaf, root, and stem of untreated banana plantlets; (**E**–**G**) are tissues of leaf, root, and stem of banana plantlets inoculated with isolate 91. Yellow arrow marks show isolate 91 cells colonized as small green dots in all tissues of a banana plant.

Biocontrol microorganisms must possess effective colonization abilities for plant disease management and growth promotion. Therefore, we assessed the colonization of GFP-tagged isolate 91 in banana tissue culture plantlets after 3 days of inoculation using confocal laser scanning microscopy (CLSM) (Figure 4). In comparison to the control (Figure 4B–D), GFP-tagged isolate 91 cells (Figure 4A) efficiently colonized all plant tissues, appearing as green spots, with a primary distribution in the root tissue, followed by the leaf and stem tissues (Figure 4E–G).

### 3.5. Isolate 91 Utilizes Various Carbon Sources

The GNIII Biolog^R^ technique was applied to study the carbon substrate utilization pattern of microorganisms. Metabolic characteristics are crucial for biocontrol microorganisms to adapt to specific environments, such as soil and plant tissues [46]. Excitingly, isolate 91 demonstrates a versatile carbon source utilization capacity, encompassing a wide spectrum of substrates. These substrates include dextrin, d-turanose, d-raffinose, d-salicin, *N*-acetyl-d-glucosamine, *N*-acetyl-d-galactosamine, *N*-acetyl neuraminic acid, d-fructose, d-galactose, 3-methyl glucose, d-fucose, l-fucose, inosine, d-sorbitol, d-mannitol, d-arabitol, myo-inositol, glycerol, d-glucose-6-PO_4_, d-fructose-6-PO_4_, d-aspartic acid, d-serine, glycyl-l-proline, l-alanine, l-arginine, l-aspartic acid, l-glutamic acid, l-histidine, l-serine, lincomycin, niaproof 4, pectin, d-gluconic acid, d-glucuronic acid, glucuronamide, mucic acid, quinic acid, tetrazolium violet, tetrazolium blue, and l-lactic acid on GNIII Biolog^R^ plate (Figure S1 and Table S2). 

## 4. Discussion

The rhizosphere, housing both beneficial and pathogenic microorganisms, acts as the primary defense against soil-borne diseases [47]. Plant growth-promoting rhizobacteria (PGPR) play a dual role in enhancing nutrient uptake by plants and acting as biocontrol agents, reducing soil-borne diseases [48]. Consequently, the screening of rhizosphere-competent bacteria represents an effective strategy for managing fungal pathogens. This study aimed to isolate highly effective antagonistic rhizobacteria against the BFW pathogen, *Foc*. A total of 109 bacterial strains were isolated from rhizospheric soil samples of banana plants in Guangxi, China. Among them, *P. aeruginosa* strain 91 exhibited the most potent antagonistic activity, prompting its selection for further investigation. Due to their proven biocontrol efficacy against various diseases, *P. aeruginosa* strains are recognized as valuable tools for disease management in tropical regions [49]. However, *P. aeruginosa* is commonly characterized as an opportunistic pathogen with a broad host range, and our understanding of *P. aeruginosa* strains remains limited. Therefore, a thorough risk assessment of isolate *P. aeruginosa* 91 will be conducted before field trials, establishing a robust risk assessment framework for biofertilizer products in the future.

PGPR support plant growth through various mechanisms, including the production of IAA, P-solubilization, secretion of siderophores, and enhancing plant resilience against biotic and abiotic stresses [50]. Siderophores improve iron acquisition and inhibit plant pathogens through iron competition [51]. Phosphate-solubilizing microorganisms make previously immobile phosphorus in the soil available to plants [52]. Several Pseudomonas strains, including *P. aeruginosa*, *P. fluorescens*, and *P. brassicacearum*, have demonstrated phosphate solubilization capabilities in previous studies [23,53,54,55,56]. IAA production has been associated with the ability of many *Pseudomonas* and other strains to promote plant growth [23,39,40,57,58]. In this study, isolate 91 produced a substantial amount of IAA, both in the presence and absence of tryptophan (Table 1). This result indicates that isolate 91 has the ability to promote plant growth, which is consistent with previous studies. Several bacterial strains produce secondary metabolites like HCN and ammonia, which play crucial roles in preventing fungal infections in various plants [19] or increasing plant nutrient availability [59]. Similarly, isolate 91 exhibited a high production of HCN and ammonia, suggesting its involvement in suppressing Foc or facilitating plant growth.

Biocontrol strategies involve competition for resources, the production of inhibitory chemicals and cell wall-degrading enzymes, and the induction of systemic resistance [60]. *Pseudomonas*, a diverse genus comprising numerous species, has been effectively employed as a plant inoculant to enhance plant growth and health [21]. While various *Pseudomonas* spp. strains have been explored as antagonists against *Foc*, most research has focused on *P. fluorescens*, with limited attention given to *P. aeruginosa* [6]. Prior studies have examined the potential of different *P. aeruginosa* strains to suppress *Foc* growth, exhibit plant growth-promoting (PGP) traits, and secrete hydrolytic enzymes in vitro [61,62,63]. An isolated strain *P. aeruginosa* BG from seawater demonstrated the ability to produce IAA (19 μg mL^−1^) and ammonia (27 μg mL^−1^), alongside the production of cyanide, iron carriers, and hydrogen peroxidase; the authors of the study believe that this strain holds significant potential for promoting plant growth and controlling plant diseases [61]. Our strain 91 exhibited higher levels of IAA and ammonia production (Table 1) compared to the marine *P. aeruginosa* BG, and it displayed similar inhibitory effects on *Foc*. Thus, it is undeniable that strain 91 has substantial application prospects. The previous research findings reveal the *P. aeruginosa* capability to produce a wide array of antifungal substances, including enzymes such as catalase, chitin-binding protein, and protease [64], alongside small molecule compounds like 3,4-dihydroxy-*N*-methyl-4-(4-oxochroman-2-yl)butanamide, pyocyanin, rhamnolipids, phenazine-1-carboxylic acid, and phenazine-1-carboxamide [65,66,67,68,69]. These components exhibit significant impacts on fungal hyphal growth, resulting in abnormal growth, unusual bending, or hyphal breakage, closely mirroring our experimental outcomes (Figure 2F). This strongly suggests that strain 91 may produce analogous compounds capable of inhibiting the growth of *Foc*.

An organism’s metabolic traits are vital for fostering plant growth and forming a successful host community [70]. Numerous *Pseudomonas* strains, including *P. aeruginosa* and *P. koreensis*, have previously showcased diverse carbon utilization patterns, contributing to enhanced plant development and fungal infection protection [23,39]. In this investigation, isolate 91 demonstrated diverse carbon utilization patterns on GNIII Biolog plate^R^ (Figure S1), highlighting its potential advantages in promoting plant growth and development. Furthermore, this observation underscores its robust adaptability to various environments and its capacity to compete effectively with other microorganisms in diverse ecological settings.

Competitive rhizosphere colonization is a crucial aspect of PGPR–plant interactions [71]. Gaining insights into the molecular mechanisms underlying banana–rhizobacteria interactions can pave the way for technical enhancements in banana cultivation. Our observation of the GFP-tagged isolate 91 exhibiting robust colonization across all tissues of cultivated banana plantlets (Figure 4) reaffirms its substantial potential for the management of BFW. On one hand, in comparison to conventional biocontrol bacteria, it appears to possess heightened efficacy in controlling BFW. On the other hand, it can more readily fulfill its function in promoting plant growth.

## 5. Conclusions

Employing efficient biocontrol bacteria with PGP traits holds promise for safeguarding crops against diseases and augmenting crop yields. This study highlights *Pseudomonas aeruginosa* 91, isolated from the banana rhizosphere, as a potent antifungal agent against *Foc*, employing a multifaceted mechanism encompassing the production of bioactive compounds and the secretion of cell wall-degrading enzymes. Isolate 91 also demonstrates diverse PGP traits, a broad carbon source utilization spectrum, and successful colonization within all banana plant tissues. In summary, isolate 91 emerges as a prospective biocontrol strain for mitigating BFW. However, its performance in controlling *Foc* and enhancing banana growth necessitates assessment through future field trials.

## Figures and Tables

**Table 1:** Functional characteristics of antifungal isolate *Pseudomonas aeruginosa* 91 isolated from the banana rhizosphere.

Parameters	*P. aeruginosa* 91
Plant Growth-Promoting Traits	
Siderophore production	85.21 ± 0.06 PSU
Phosphate solubilization	1.38 ± 0.03 PSI
Ammonia production	5.53 ± 0.07 µmoL mL^−1^
HCN production	+
1-Aminocyclopropane-1-carboxylic deaminase activity	519.28 ± 6.58 nmoL α-ketobutyrate mg^−1^ h^−1^
Indole Acetic Acid (µg mL^−1^)	
Absence of Tryptophan	49.53 ± 1.48
Presence of Tryptophan (0.5 %)	175.96 ± 1.63
Hydrolytic Enzyme Production (IU mL^−1^)	
Cellulase	451.23 ± 5.37
Protease	143.56 ± 1.82
Chitinase	495.43 ± 5.32
Glucanase	665.76 ± 10.84

PSU, percent siderophore unit; PSI, phosphate solubilization index; +, positive.

## Data Availability

The data presented in this study are available in the article.

## Supplementary Materials

The following supporting information can be downloaded at: https://www.mdpi.com/article/10.3390/jof9111047/s1, Table S1: Media used in this study; Table S2: List of carbon sources utilized by *Pseudomonas aeruginosa* 91 in GENIII BIOLOGR plate; Figure S1: Carbon substrate utilization pattern of isolate 91 on GNIII Biolog^R^ plate.
