# Supplementary material for: Pseudomonas aeruginosa Strain 91: A Multifaceted Biocontrol Agent against Banana Fusarium Wilt
Source: J Fungi (Basel). 2023 Oct 25;9(11):1047. doi: 10.3390/jof9111047 (PMC10672659; doi:10.3390/jof9111047)
Supplement: Supplementary file 1 [file jof-09-01047-s001.zip › jof-2682357-supplementary.pdf]

Table S1. Media used in this study.

Media Name	Composition	Quantity
**Nutrient Agar**	Peptone	5 g
	Beef extract	3 g
	Sodium chloride	5 g
	Agar	18 g
	Ultrapure water	1000 mL
**Potato Dextrose Agar**	Potato	200 g
	Dextrose	20 g
	Agar	18 g
	Ultrapure water	1000 mL
**Luria Bertani Agar**	Yeast extract	5 g
	Peptone	10 g
	NaCl	10 g
	Agar	18 g
	Ultrapure water	1000 mL
**Dworkin and Foster Salts Minimal Medium**	Glucose	2 g
	Gluconic acid	2 g
	Citric acid	2 g
	KH_2PO_4	4 g
	Na_2HPO_4	6 g
	$MgSO_4.7H_2O$	0.2 g
	$CaCl_2$	0.2 g

	$FeSO_4.7H_2O$	0.2 g
	H_3BO_3	0.015 g
	$ZnSO_4.7H_2O$	0.02 g
	Na_2MoO_4	0.01 g
	KI	0.01 g
	NaBr	0.01 g
	$MnCl_2$	0.01 g
	$COCl_2$	0.005 g
	$CuCl_2$	0.005 g
	$AlCl_3$	0.002 g
	$NiSO_4$	0.002 g
	Ultrapure water	1000 mL
**Yeast Mannitol Agar**	Mannitol	15 g
	K_2HPO_4	0.5 g
	Yeast extract	0.4 g
	$MgSO_4.7H_2O$	0.2 g
	NaCl	0.1 g
	Agar	15 g
	Ultrapure water	1000 mL
**Pikovskaya's Agar**	Yeast extract	0.5 g
	Dextrose	10 g
	Calcium phosphate	5 g
	Ammonium sulphate	0.5 g
	Potassium chloride	0.2 g

	Magnesium sulphate	0.1 g
	Manganese sulphate	0.0001 g
	Ferrous sulphate	0.0001 g
	Agar	15 g
	Ultrapure water	1000 mL
**Yeast Extract Medium**	Yeast extract	5 g
	Dextrose	20 g
	Agar	18 g
	1000×trace element	1 mL
**1000×Trace Element**	$ZnSO_4.7H_2O$	2.2 g
	H_3BO_3	1.1 g
	$MnCl_2.4H_2O$	0.5 g
	$FeSO_4.7H_2O$	0.5 g
	$CoCl_2.5H_2O$	0.16 g
	$CuSO_4$	0.16 g
	$(NH_4)_6Mo_7O_{24}.4H_2O$	0.11 g
	EDTA	5.0 g
	Ultrapure water	100 mL

Table S2. List of carbon sources utilized by *Pseudomonas aeruginosa* 91 in GENIII BIOLOG^R plate.

**S. No.**	**Carbon Source**
1	Dextrin
2	D-Turanose
3	D-Raffinose
4	D-Salicin
5	N-Acetyl-D-Glucosamine
6	N-Acetyl-D-Galactosamine
7	N-AcetylNeuraminic Acid
8	D-Fructose
9	D-Galactose
10	3-Methyl Glucose
11	D-Fucose
12	L-Fucose
13	Inosine
14	D-Sorbitol
15	D-Mannitol
16	D-Arabitol
17	myo-Inositol
18	Glycerol
19	D-Glucose-6-PO_4
20	D-Fructose-6-PO_4
21	D-Aspartic Acid

22	D-Serine
23	Glycyl-L-Proline
24	L-Alanine
25	L-Arginine
26	L-Aspartic Acid
27	L-Glutamic Acid
28	L-Histidine
29	L-Serine
30	Lincomycin
31	Niaproof 4
32	Pectin
33	D-Gluconic Acid
34	D-Glucuronic Acid
35	Glucuronamide
36	Mucic Acid
37	Quinic Acid
38	Tetrazolium Violet
39	Tetrazolium Blue
40	L-Lactic Acid

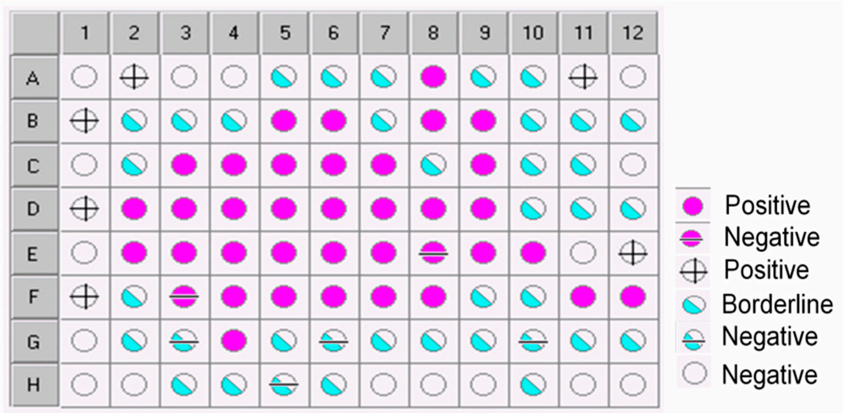

Figure S1. Carbon substrate utilization pattern of isolate 91 on GNIII Biolog[R] plate.
